# Dieckol, an Algae-Derived Phenolic Compound, Suppresses UVB-Induced Skin Damage in Human Dermal Fibroblasts and Its Underlying Mechanisms

**DOI:** 10.3390/antiox10030352

**Published:** 2021-02-26

**Authors:** Lei Wang, Jun-Geon Je, Hye-Won Yang, You-Jin Jeon, Seungheon Lee

**Affiliations:** 1 College of Food Science and Engineering, Ocean University of China, Qingdao 266003, China; comeonleiwang@163.com; 2 Department of Marine Life Sciences, Jeju National University, Jeju 63243, Korea; wpwnsrjs@naver.com (J.-G.J.); koty221@naver.com (H.-W.Y.); 3 Marine Science Institute, Jeju National University, Jeju 63333, Korea

**Keywords:** UVB irradiation, Dieckol, photoaging, matrix metalloproteinases, pro-inflammatory cytokines

## Abstract

Ultraviolet (UV) irradiation is considered to be the primary environmental factor that causes skin damage. In the present study, we investigated the protective effect of dieckol (DK), a compound isolated from the brown seaweed *Ecklonia cava*, against UVB-induced skin damage in human dermal fibroblasts (HDF cells). The results indicated that DK effectively inhibited the activity of collagenase. DK remarkably reduced the intracellular reactive oxygen species level and improved the viability of UVB-irradiated HDF cells. Besides, DK significantly and dose-dependently improved collagen synthesis and inhibited intracellular collagenase activity in UVB-irradiated HDF cells. In addition, DK markedly reduced the expression of proinflammatory cytokines and matrix metalloproteinases. Further analyses revealed that these processes were mediated through the regulation of nuclear factor kappa B, activator protein 1, and mitogen-activated protein kinase signaling pathways in the UVB-irradiated HDF cells. In conclusion, these results indicate that DK possesses strong in vitro photoprotective effects and therefore has the potential to be used as an ingredient in the cosmeceutical industry.

## 1. Introduction

Skin is the largest organ in the human body and also directly exposed to the outside environment. The skin is constantly exposed to solar ultraviolet (UV) irradiation during normal daily life activities. Overexposure to UV irradiation leads to skin damage and diseases, such as edema, hyperpigmentation, erythema, sunburn, and cancer [1,2]. UVB (280–320 nm) irradiation, a subtype of UV irradiation, induces more stress in humans than the other UV subtypes [3]. Previous studies have reported UVB-induced skin diseases and damage mainly by stimulating the expression of proinflammatory cytokines and matrix metalloproteinases (MMPs) by inducing overproduction of intracellular reactive oxygen species (ROS) [4,5]. Therefore, an antioxidant agent that possesses strong ROS scavenging effect may be a potential candidate for the development of a therapeutic drug or cosmetic for the treatment of UVB-induced skin damage. Given that natural products have several advantages such as strong activity and low or no adverse effects, the discovery of ingredients from natural resources has attracted considerable research interest.

**Figure 1:**
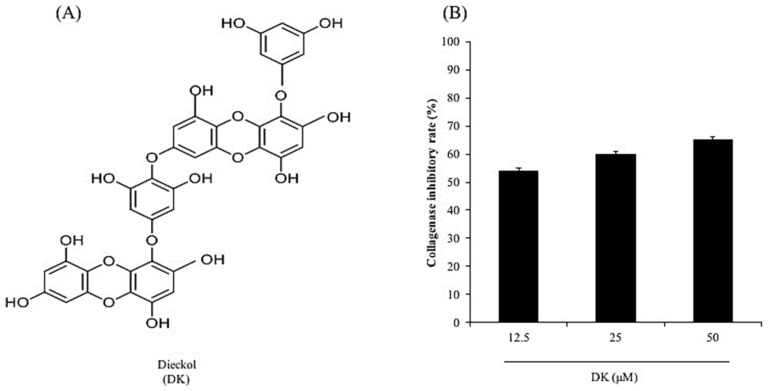
DK inhibits collagenase activity. (**A**) Structure of DK. (**B**) Inhibitory effect of DK on collagenase.

Seaweeds are rich in phenolic compounds, pigments, minerals, fatty acids, amino acids, and polysaccharides [6]. Several seaweed-derived compounds have been reported to have considerable health benefits through their antioxidant, antibacterial, anti-allergic, anti-hypertensive, anti-obesity, anti-inflammatory, anticancer, and photoprotective activities [7,8,9,10,11,12,13]. Dieckol (DK, Figure 1A) is a phenolic compound isolated from the brown seaweed *Ecklonia cava*. Numerous studies have reported that DK possesses various health benefits in humans [14,15,16,17]. In addition, previous studies have investigated the effect of DK on UVB-induced skin damage [18,19,20]. Ko et al. (2011) investigated the protective effect of DK on UVB-induced oxidative stress in vitro in human keratinocytes (HaCaT cells) and in vivo in zebrafish. The results suggested that DK effectively protected HaCaT cells and zebrafish against oxidative stress induced by UVB irradiation [20]. Cha et al. (2012) evaluated the effect of DK on UVB-induced melanogenesis in zebrafish. The results indicated that DK significantly and dose-dependently reduced melanin synthesis in UVB-irradiated zebrafish [19]. These results demonstrate that DK effectively suppresses UVB-induced skin damage and suggest the potential application of DK in cosmetics. However, the effect of DK on UVB-induced skin wrinkling has not yet been investigated. To further explore the potential of DK in cosmetics, the effects of DK on UVB-induced skin wrinkling and its underlying mechanism were investigated in human dermal fibroblasts (HDF cells) in the present study.

## 2. Materials and Methods

### 2.1. Chemicals and Reagents

Dimethyl sulfoxide (DMSO), 3-(4-5-dimethyl-2yl)-2-5-diphynyltetrasolium bromide (MTT), collagenase, azo dye-impregnated collagen, and 2′, 7- dichlorodihydrofluorescein diacetate (DCFH2-DA) were purchased from Sigma Co. (St. Louis, MO, USA). Phosphate-buffered saline (PBS), penicillin/streptomycin (P/S), Dulbecco’s modified Eagle’s medium (DMEM), F-12 medium, and fetal bovine serum (FBS) were purchased from Gibco BRL (Life Technologies, Burlington, ON, Canada). Antibodies against p-p38, p-JNK, p-ERK, GAPDH, p-c-Jun, nucleolin, NF-κB p65, and NF-κB p50 were purchased from Cell Signaling Technology (Beverly, MA, USA). The ELISA kits used for analysis of human MMPs, interleukin-6 (IL-6), interleukin-1 beta (IL-1β), and tumor necrosis factor-alpha (TNF-α) were purchased from GE Healthcare Life Sciences (Exeter, Devon, UK). The PIP EIA kit was purchased from TaKaRa Bio Inc. (Kusatsu, Japan). All other chemicals used in this study were of analytical grade.

### 2.2. Preparation of DK

DK was prepared according to the protocol described in a previous study [21]. In brief, *E. cava* was extracted with 70% ethanol. The ethanol extract of *E. cava* was fractionated using hexane, chloroform, and ethyl acetate. The ethyl acetate fraction was separated by preparative centrifugal partition chromatography (CPC), and the DK-enriched fraction was further purified by high-performance liquid chromatography (HPLC) until its purity was higher than 95%. Finally, DK was identified and confirmed by HPLC–DAD–ESI/MS [21]. DK was stored at −20 °C and confirmed the purity before using.

### 2.3. Measurement of Collagenase Activity

The inhibitory effect of DK on collagenase from *Clostridium histolyticum* was evaluated according to the protocol described by Wang et al. [22]. The collagenase inhibitory rates were calculated based on the control (distilled water, 0%).

### 2.4. Cell Culture and UVB Irradiation

HDF cells (ATCC^®^ PCS20101™) were cultured in a mixed medium (DMEM: F-12, 3:1) supplemented with 1% P/S and 10% FBS. Cells were plated at a concentration of 3 × 10^4^ cells/mL for the experiments. In the previous study, the viabilities of HDF cells irradiated with several dose of UVB were evaluated and the results indicated that 50 mJ/cm^2^ of UVB decreased the viability of HDF cells by 50%. Thus, 50 mJ/cm^2^ of UVB was applied to HDF cells in this study [22]. Cells were irradiated with UVB (50 mJ/cm^2^) in PBS and the UVB-irradiated cells were then incubated with FBS-free medium until analysis.

### 2.5. Determination of Intracellular ROS Level and Cell Viability

In our previous study, the viabilities of HDF cells treated with different concentrations of DK were evaluated and the results indicated that DK was non-toxic to HDF cells at the concentration under 50 μM. Therefore, 50 μM was decided as the maximum concentration applied to HDF cells [23]. HDF cells were plated in a 24-well plate and incubated for 24 h. To measure intracellular ROS level in the UVB-irradiated HDF cells, cells were treated with different concentrations of DK (12.5, 25, and 50 μM) for 30 min. The DK-treated cells were incubated with DCFH2-DA (0.5 mg/mL stock solution) for 30 min. Then, the cells were irradiated with UVB and the fluorescence intensity of the cells were detected using a microplate reader [5]. To evaluate the viability of the UVB-irradiated HDF cells, the cells were plated and treated with DK. After 2 h, the DK-treated HDF cells were irradiated with UVB and incubated with FBS-free medium for 48 h. The viability of UVB-irradiated HDF cells was assessed by MTT assay according to the protocol described by Wang et al. [23].

### 2.6. Evaluation of Intracellular Collagenase Activity

HDF cells were plated in a 100 mm cell culture dish and incubated for 24 h. Cells were treated with DK for 2 h and irradiated with UVB. The UVB-irradiated HDF cells were further incubated for 48 h and then harvested. Cells were lysed and intracellular collagenase activity in the cells was evaluated according to the method described in previous studies [22,24]. The intracellular collagenase activity of HDF cells without UVB irradiation and DK treatment (control group) was referred as 100%. The intracellular collagenase activities of UVB-irradiated or DK-treated HDF cells were calculated based on the control group (100%).

### 2.7. Enzyme-Linked Immunosorbent Assay

HDF cells were treated with DK for 2 h and irradiated with UVB. After 48 h, the cell culture medium was collected to measure the collagen, proinflammatory cytokines, and MMP levels. The relative amounts of collagen, proinflammatory cytokines, and MMPs in the UVB-irradiated HDF cells were evaluated using ELISA kits according to the manufacturer’s instructions.

### 2.8. Western Blot Analysis

HDF cells were treated with DK for 2 h and irradiated with UVB. After 1 h of incubation, the UVB-irradiated HDF cells were harvested, lysed, and the proteins were extracted. The amounts of activated NF-κB, AP-1, and MAPKs proteins were evaluated by Western blot analysis [22].

### 2.9. Statistical Analysis

Experiments were performed in triplicate and data are expressed as the mean ± standard error (SE). One-way ANOVA was used to compare the means of each treatment using SPSS 17.0. Significant differences between the means were identified by Duncan’s test.

## 3. Results

### 3.1. DK Suppresses Dermic Damage Induced by UVB Irradiation

As shown in Figure 1B, the collagenase inhibitory rates of DK at concentrations of 12.5, 25, and 50 μM were 53.94, 59.82, and 64.95%, respectively. It displays that DK effectively inhibited collagenase in a dose-dependent manner.

**Figure 2:**
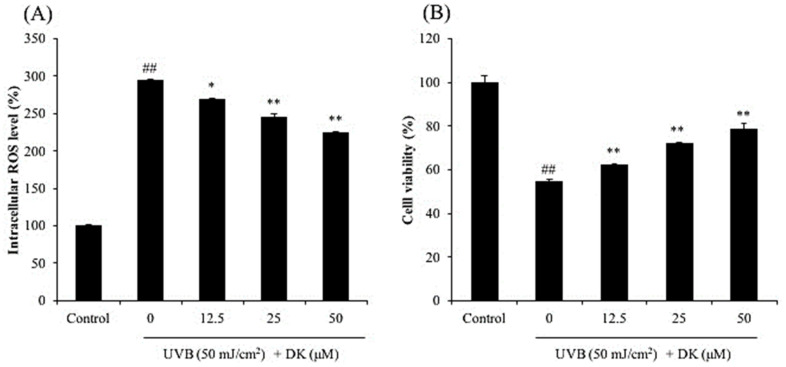
Protective effect of DK against UVB-induced damage in HDF cells. (**A**) Intracellular ROS scavenging effect of DK in UVB-irradiated HDF cells, and (**B**) protective effect of DK on UVB-induced cell death in HDF cells. Cell viability was measured by MTT assay and intracellular ROS level was measured by DCF-DA assay. The data were expressed as the mean ± SE (*n* = 3). * *p* < 0.05, ** *p* < 0.01 as compared to the UVB-irradiated group, and ## *p* < 0.01 as compared to the control group.

As shown in Figure 2A, intracellular ROS level in UVB-irradiated cells was significantly increased to 294.45% compared to the control group (100%). However, the intracellular ROS levels in cells treated with 12.5, 25, and 50 μM DK decreased to 268.56, 245.75, and 223.80%, respectively (Figure 2A). As shown in Figure 2B, the viability of UVB-irradiated HDF cells was markedly decreased to 54.64% compared to that of the control group (100%). However, the viability of UVB-irradiated HDF cells treated with 12.5, 25, and 50 μM DK increased to 62.19, 71.96, and 78.74%, respectively (Figure 2B). These results indicated that DK protected HDF cells against UVB-induced cell death through ROS scavenging.

**Figure 3:**
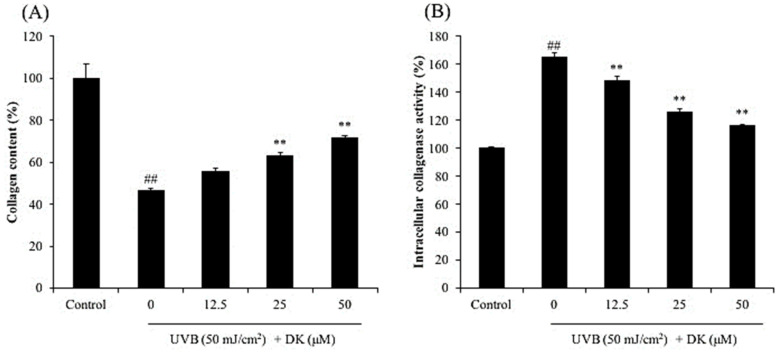
DK improves collagen synthesis and inhibits intracellular collagenase activity in UVB-irradiated HDF cells. (**A**) The effect of DK on collagen synthesis in UVB-irradiated HDF cells. (**B**) The effect of DK on intracellular collagenase activity in UVB-irradiated HDF cells. The data were expressed as the mean ± SE (*n* = 3). ** *p* < 0.01 as compared to the UVB-irradiated group, and ## *p* < 0.01 as compared to the control group.

UVB irradiation not only inhibits collagen synthesis, but also degrades collagen by stimulating the activity of collagenase in dermic fibroblasts, which consequently causes wrinkle formation [22,25]. To investigate the effect of DK on UVB-induced skin wrinkling, the collagen synthesis level and relative intracellular collagenase activity were assessed in UVB-irradiated HDF cells. As shown in Figure 3A, collagen synthesis level in UVB-irradiated HDF cells was significantly reduced compared to that in the non-irradiated cells. However, the collagen synthesis level in DK-treated HDF cells was markedly increased in a dose-dependent manner (Figure 3A). In addition, the intracellular collagenase activity was increased in the cells irradiated with UVB, however, decreased in the cells treated with DK (Figure 3B). These results demonstrate that DK not only protects the skin against UVB-mediated inhibition of collagen synthesis, but also inhibits collagen degradation by reducing the relative intracellular collagenase activity in UVB-irradiated HDF cells.

### 3.2. DK Reduces the Production of MMPs and Pro-Inflammatory Cytokines in UVB-Irradiated HDF Cells

**Figure 4:**
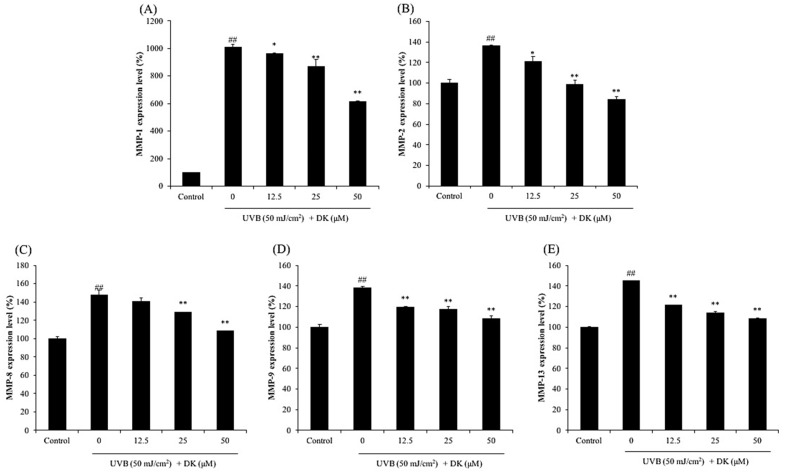
DK inhibits MMPs expression in UVB-irradiated HDF cells. (**A**) MMP-1 expression level of UVB-irradiated HDF cells. (**B**) MMP-2 expression level of UVB-irradiated HDF cells. (**C**) MMP-8 expression level of UVB-irradiated HDF cells. (**D**) MMP-9 expression level of UVB-irradiated HDF cells. (**E**) MMP-13 expression level of UVB-irradiated HDF cells. The data were expressed as the mean ± SE (*n* = 3). * *p* < 0.05, ** *p* < 0.01 as compared to the UVB-irradiated group, and ## *p* < 0.01 as compared to the control group.

**Figure 5:**
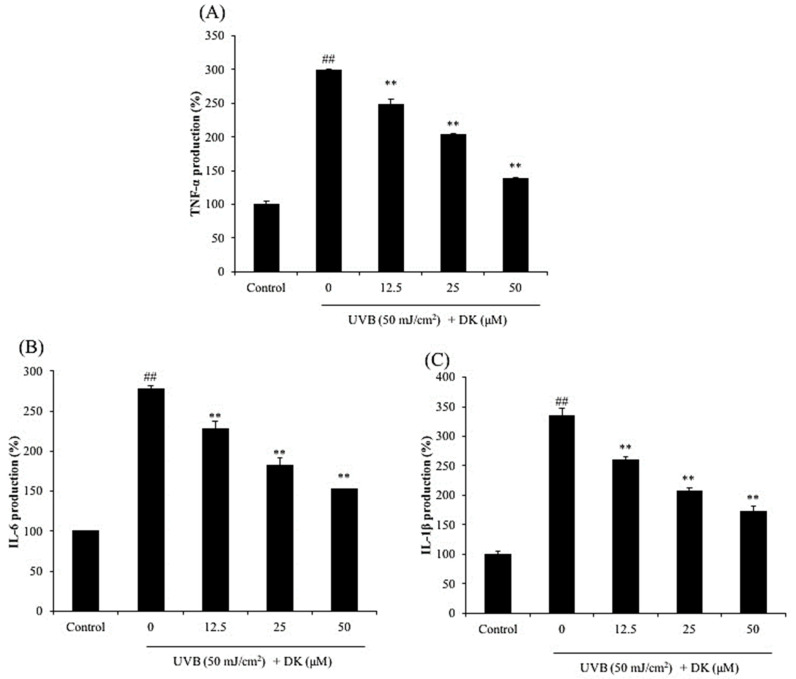
DK suppresses the expression of pro-inflammatory cytokines in UVB-irradiated HDF cells. (**A**) TNF-α level in UVB-irradiated HDF cells. (**B**) IL-6 level in UVB-irradiated HDF cells. (**C**) IL-1β level in UVB-irradiated HDF cells. The pro-inflammatory cytokines levels were measured using the ELISA kits. The data were expressed as the mean ± SE (*n* = 3). ** *p* < 0.01 as compared to the UVB-irradiated group, and ## *p* < 0.01 as compared to the control group.

As shown in Figure 4, the levels of MMPs including MMP-1, 2, 8, 9, and 13 of HDF cells irradiated by UVB were significantly increased compared to non-irradiated cells. However, the levels of MMPs were remarkably and dose-dependently reduced in DK-treated cells. In addition, UVB significantly increased the expressions of pro-inflammatory cytokines including TNF-α, IL-6, and IL-1β (Figure 5), whereas DK effectively decreased the levels of these pro-inflammatory cytokines in a dose-dependent manner. These results indicate that DK effectively inhibited collagen degradation and inflammatory response stimulated by UVB irradiation by reducing the expression of MMPs and pro-inflammatory cytokines in HDF cells.

### 3.3. DK Regulates NF-κB, AP-1, and MAPKs Pathways in UVB-Irradiated HDF Cells

**Figure 6:**
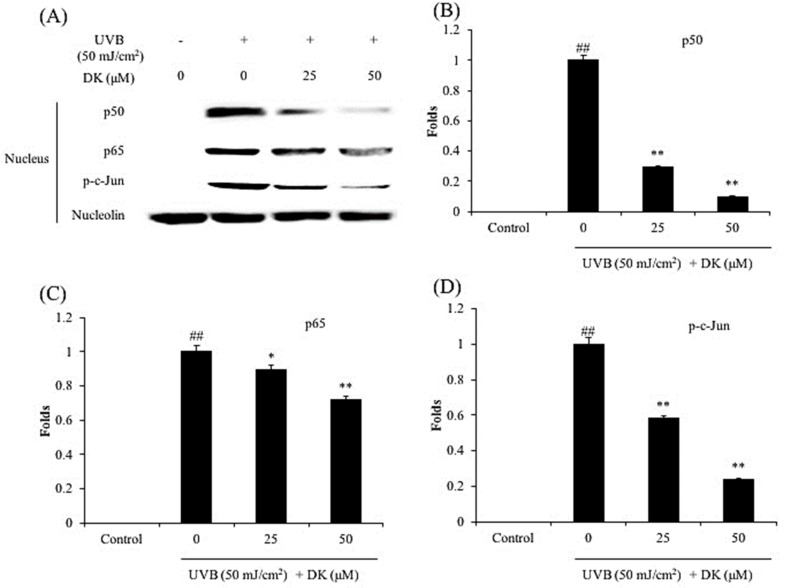
DK inhibits NF-κB activation and AP-1 phosphorylation in UVB-irradiated HDF cells. (**A**) The nuclear NF-κB-related proteins and phosphorylated AP-1 (p-c-Jun) levels in UVB-irradiated HDF cells, (**B**) the relative amount of NF-κB p50 in UVB-irradiated HDF cells, (**C**) the relative amount of NF-κB p65 in UVB-irradiated HDF cells, and (**D**) the relative amount of p-c-Jun in UVB-irradiated HDF cells. The relative amounts of NF-κB p65, NF-κB p50, and p-c-Jun levels were compared with nucleolin. The data were expressed as the mean ± SE (*n* = 3). * *p* < 0.05, ** *p* < 0.01 as compared to the UVB-irradiated group, and ## *p* < 0.01 as compared to the control group.

The effect of DK on NF-κB, AP-1, and MAPKs pathways in UVB-irradiated HDF cells was investigated by Western blot. As shown in Figure 6, DK significantly reduced nuclear p50 and p65 levels in UVB-irradiated HDF cells. In addition, the phosphorylated c-Jun level was significantly increased by UVB irradiation (Figure 6 ). However, it effectively and dose-dependently reduced by DK treatment (Figure 6). These results indicate that DK could effectively regulates NF-κB and AP-1 pathways in UVB-irradiated HDF cells. 

**Figure 7:**
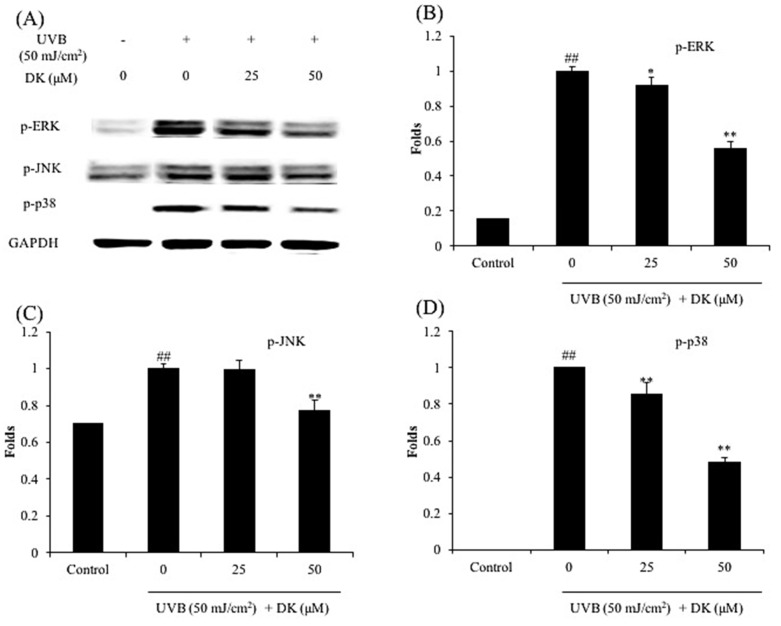
DK suppresses the activation of MAPKs in UVB-irradiated HDF cells. (**A**) The levels of active MAPKs (p-ERK, p-JNK, and p-p38) in UVB-irradiated HDF cells, (**B**) the relative amount of p-ERK in UVB-irradiated HDF cells, (**C**) the relative amount of p-JNK in UVB-irradiated HDF cells, and (**D**) the relative amount of p-p38 in UVB-irradiated HDF cells. The relative amounts of activated MAPKs levels were compared with GAPDH. The data were expressed as the mean ± SE (*n* = 3). * *p* < 0.05, ** *p* < 0.01 as compared to the UVB-irradiated group, and ## *p* < 0.01 as compared to the control group.

Furthermore, UVB significantly increased the level of activated MAPKs (p-JNK, p-ERK, and p-p38) (Figure 7). In contrast, the levels of activated MAPKs were markedly and dose-dependently decreased in cells treated with DK (Figure 7). These results demonstrate that DK attenuates UVB-induced MMP and pro-inflammatory cytokine expression in HDF cells by regulating AP-1, NF-κB, and MAPKs pathways.

In summary, the above results indicate that DK protected skin against UVB irradiation-induced dermic damage in HDF cells. DK exerted its protective effects in the following ways: improved cell viability via scavenging of intracellular ROS, restored collagen synthesis, and reduced collagen degradation by inhibition of intracellular collagenase activity, and attenuation of MMP and proinflammatory cytokine expression through regulation of the NF-κB, AP-1, and MAPKs pathways.

## 4. Discussion

Collagen is the main structural protein of the extracellular matrix. Degradation of collagen decreases the thickness of the skin and causes wrinkle formation [22]. Collagenase is the enzyme that degrades collagen. Thus, a collagenase inhibitor may be an ideal candidate to suppress skin wrinkling by reducing collagen degradation. In the present study, the inhibitory effect of DK on commercial collagenase was investigated. The result indicates that DK effectively inhibited collagenase activity in a dose-dependent manner. It suggests that DK may possess the potential to suppress skin wrinkling by inhibiting collagen degradation via inhibition of collagenase. Therefore, in further studies, the protective effect of DK on UVB-induced dermic damage was investigated in HDF cells. The protective effect of DK against UVB irradiation-induced dermic damage was investigated by evaluating the intracellular ROS level, cell viability, collagen synthesis level, and intracellular collagenase activity in UVB-irradiated HDF cells. The results indicate that DK effectively protects HDF cells against oxidative damage in a dose-dependent manner.

UVB irradiation not only inhibits collagen synthesis, but also degrades collagen by stimulating the activity of collagenase in dermic fibroblasts, which consequently causes wrinkle formation [22,25]. To investigate the effect of DK on UVB-induced skin wrinkling, the collagen synthesis level and relative intracellular collagenase activity were assessed in UVB-irradiated HDF cells. As shown in Figure 3A, collagen synthesis level in UVB-irradiated HDF cells was significantly reduced compared to that in the non-irradiated cells. However, the collagen synthesis level in DK-treated HDF cells was markedly increased in a dose-dependent manner (Figure 3A). These results demonstrate that DK not only protects the skin against UVB-mediated inhibition of collagen synthesis, but also inhibits collagen degradation by reducing the relative intracellular collagenase activity in UVB-irradiated HDF cells.

MMPs are a family of proteases comprising of several enzymes including collagenases that degrade various types of collagens and have been implicated in various diseases [26,27,28]. MMP-1 plays an important role in skin wrinkling owing to its ability to degrade the major structural proteins of skin including type I and type III collagen [22]. Therefore, MMP-1 has been a target for studies on cosmetics. Pro-inflammatory cytokines play an important role in human health because they are associated with various diseases, especially inflammation [29,30]. In addition, various reports suggest that pro-inflammatory cytokines regulate the expression of MMPs. Therefore, the effect of DK on the expression of MMPs and pro-inflammatory cytokines in UVB-irradiated HDF cells was investigated in the present study.

The expression of MMP-1, -2, -8, -9, and -13 was evaluated in HDF cells irradiated with UVB with/without treatment with DK. As shown in Figure 4, UVB irradiation significantly increased the expression of MMPs. The expression of MMP-1 in HDF cells was increased by 9-fold following UVB irradiation compared to that in the control cells (Figure 4A). However, the expression of MMPs in UVB-irradiated HDF cells were effectively and dose-dependently suppressed by DK treatment (Figure 4). As shown in Figure 5, the expression of pro-inflammatory cytokines, including TNF-α, IL-6, and IL-1β, was significantly elevated in HDF cells following UVB irradiation. However, DK markedly attenuated the expression of these pro-inflammatory cytokines in the UVB-irradiated HDF cells in a dose-dependent manner. These results indicate that DK effectively inhibited collagen degradation and inflammatory response stimulated by UVB irradiation by reducing the expression of MMPs and pro-inflammatory cytokines in HDF cells.

Previous reports suggest that UVB-stimulated the expression of MMPs and pro-inflammatory cytokines through activating AP-1, NF-κB, and MAPK pathways, and the activation of these pathways is inhibited by natural compounds through the scavenging of intracellular ROS [5,22,31,32,33]. MAPKs, including JNK, ERK, and p38, regulate various cellular functions and are activated by various stimuli such as cytokines and ROS. Phosphorylated MAPKs translocate to the nucleus and stimulate the activation of AP-1 that then causes upregulation of MMP expression [32]. The production of pro-inflammatory cytokines is mainly regulated by the NF-κB pathway. Activated NF-κB molecules translocate to the nucleus and stimulate pro-inflammatory cytokine expression [5]. To investigate the mechanism of inhibition of DK on UVB-stimulated MMP and pro-inflammatory cytokine expression, the levels of activated AP-1, NF-κB, and MAPKs were assessed in UVB-irradiated HDF cells.

As shown in Figure 6, the levels of the nuclear NF-κB subunits (p50 and p65) were significantly increased following UVB irradiation of HDF cells. However, both p50 and p65 protein levels in the nucleus were markedly reduced following DK treatment of the UVB-irradiated HDF cells (Figure 6A–C). In addition, UVB irradiation significantly increased the level of activated AP-1 (p-c-Jun) in HDF cells (Figure 6A,D). However, p-c-Jun levels were dose-dependently reduced following treatment of the cells with DK (Figure 6A,D). Furthermore, UVB significantly increased the level of activated MAPKs (p-JNK, p-ERK, and p-p38) (Figure 7). In contrast, the levels of activated MAPKs were markedly and dose-dependently decreased in cells treated with DK. These results demonstrate that DK attenuates UVB-induced MMP and pro-inflammatory cytokine expression in HDF cells by regulating AP-1, NF-κB, and MAPKs pathways.

In summary, the above results indicate that DK protected skin against UVB irradiation-induced dermic damage in HDF cells. DK exerted its protective effects in the following ways: improved cell viability via scavenging of intracellular ROS; restored collagen synthesis; reduced collagen degradation by inhibition of intracellular collagenase activity; and attenuation of MMP and pro-inflammatory cytokine expression through regulation of the NF-κB, AP-1, and MAPKs pathways.

## 5. Conclusions

In the present study, the protective effect of the algae-derived phenolic compound, DK, on UVB-induced photoaging was investigated in HDF cells. The results indicated that DK effectively protected against UVB-induced damage in HDF cells. In conclusion, this study suggests that DK possesses strong photoprotective effects and may potentially be used as an active ingredient to develop a cosmetic or a medicine to protect skin against photoaging in the cosmetic and pharmaceutical industries. However, further clinical study is essential to develop DK as a pharmaceutical or a cosmetic ingredient for counteracting photodamage induced by UV irradiation.

## Data Availability

All data are contained within the article.
